# Removal of intracardiac bone cement embolism after percutaneous kyphoplasty: A case report

**DOI:** 10.1097/MD.0000000000019354

**Published:** 2020-03-13

**Authors:** Yu Song, Xiaofan Huang, Long Wu

**Affiliations:** Department of Cardiovascular Surgery, Union Hospital, Tongji Medical College, Huazhong University of Science and Technology, Wuhan, China.

**Keywords:** bone cements, cardiac, foreign bodies, kyphoplasty, tricuspid valve

## Abstract

**Rationale:**

Leakage of bone cement is a common complication after percutaneous kyphoplasty. In rare cases, bone cement can leak into the venous system, which can be life threatening, especially when it causes an embolism in the heart.

**Patient concerns:**

A 79-year-old female patient developed chest pain with chest tightness 3 weeks after the percutaneous kyphoplasty.

**Diagnoses:**

Initially, negative fluoroscopy results and elevated myocardial enzymes suggested that the patient's chest pain and chest tightness symptoms were manifestations of coronary heart disease. However, in the subsequent computed tomography (CT) examination, foreign bodies in the heart and pulmonary vessels were found.

**Interventions:**

The patient underwent emergency surgery to remove the bone cement and repair the tricuspid valve.

**Outcomes:**

The postoperative course was uneventful and the patient was discharged on the 13th day after surgery.

**Lessons:**

If a patient develops chest pain with chest tightness after percutaneous kyphoplasty, the clinicians must be vigilant and take into account the limited sensitivity of fluoroscopy and use chest computer tomography and echocardiogram as the first choice and thereby prevent serious consequences.

## 1 Introduction

Percutaneous kyphoplasty (PKP) is currently the most commonly used, minimally invasive technique for treating osteoporotic vertebral fractures and osteolytic metastases. With vertebroplasty, the compressed vertebral body is first replaced by a special balloon, and the bone cement is then injected under low pressure into the damaged vertebral body to create more vertebrae stability, prevent collapse, relieve pain, and restore height to the spinal column. Although this technique has gradually been introduced and is widely used clinically, studies have reported a growing number of associated complications. The most frequent complication associated with this treatment is bone cement leakage. Cement leakage into the surrounding tissues might be asymptomatic, but could potentially cause spinal canal stenosis or compression of the nerve root. In addition, cement leakage into the perivertebral venous system can result in pulmonary and intracardiac embolisms, which can cause life-threatening complications. In this report, we present a case in which an embolism was caused by intracardiac cement leakage that resulted in severe tricuspid regurgitation and the puncture of the right ventricle.^[1–3]^

## 2 Case report

**Figure 1:**
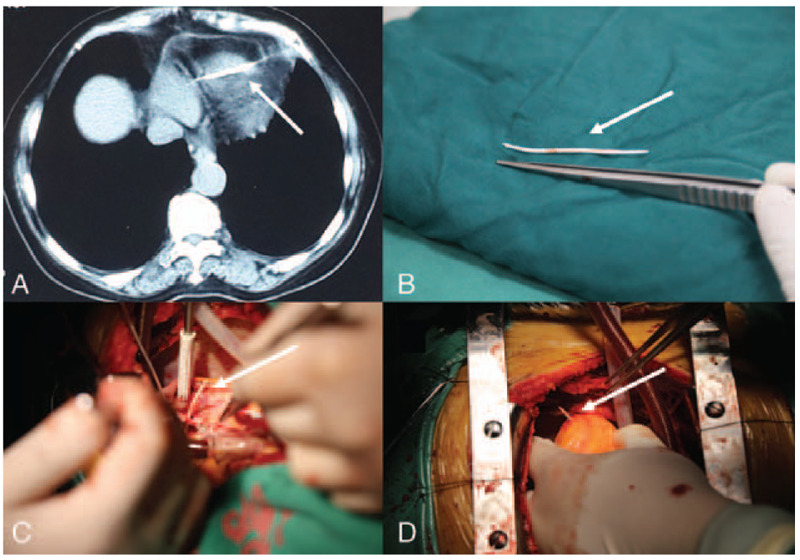
(A) Chest computed tomography showing a high-density shadow extending from the bottom of the heart to the anterior chest wall (indicated by arrow). (B) Foreign body after removal with forceps for scale. (C) Foreign body piercing the posterior flap of the tricuspid valve (indicated by the arrows). (D) Foreign body penetrating the anterior wall of the right ventricle (indicated by the arrows).

A 79-year-old female patient with hypertension presented with a compression fracture of the L5 vertebral body. The orthopedic surgeons performed PKP. Three weeks later, the patient developed chest pain with chest tightness. An electrocardiogram showed signs of myocardial ischemia. Chest tightness and tachycardia were relieved after the administration of oral antithrombotic clopidogrel; however, the patient still felt occasional chest pain. No heart murmur was observed in the physical examination, the chest x-rays suggested only an enlargement of the lung texture, and the laboratory tests showed abnormally elevated levels of myocardial enzymes; therefore, we suspected that the patient might have had coronary atherosclerotic heart disease. To determine the status of the patient's coronary artery, a coronary computed tomography angiography (CCTA) was conducted. The CCTA results indicated a high-density shadow extending from the bottom of the heart to the anterior chest wall and multiple areas of coronary stenosis. A computed tomography (CT) examination was conducted and the results revealed a mottled and striated high-density shadow in the right lower lung and a striated high-density shadow at the base of the heart (Fig. 1A). Because of the recent surgery, the possibility that this foreign body was bone cement was considered; consequently, the patient was immediately transferred to the department of cardiovascular surgery. An echocardiogram revealed a left ventricular ejection fraction of 60%, a nondilated atria, ventricles with normal systolic function, and a strong echo ∼6.8 cm long in the right ventricle, which penetrated through the tricuspid valve into the right ventricle. This type of injury leads to severe tricuspid insufficiency, with a large number of reflux signals in the tricuspid orifice and a small volume of pericardial effusion in the pericardial cavity. The patient underwent a coronary angiography and emergency surgery in the digital subtraction angiography (DSA) operating room on the same day. The coronary angiography suggested that there were multiple areas of coronary stenosis. A standard median sternotomy was performed. A cardiopulmonary bypass was established with the ascending aorta and bicaval cannulations. St. Thomas II solution was administered antegrade to induce cardioplegia following aortic cross clamping. The tricuspid valve was exposed through an oblique right atrial incision. Valve anatomy and regurgitation mechanisms were evaluated using a saline injection test. The cardiopulmonary bypass time was 86 minutes. During surgery, ∼100 mL bloody pericardial effusion was observed. The foreign body formed a needle-shaped foreign body that measured ∼8.0 × 0.3 cm. The majority of the foreign body was located in the right ventricle, piercing the posterior leaflet of the tricuspid valve and resulting in severe tricuspid regurgitation. The foreign body also pierced the anterior wall of the right ventricle and protruded from the apex cordis by ∼1.5 cm. The chordae of anterior leaflet and septal leaflet ruptured, and the ventricular surface of the septum had the appearance of jelly-like fine-grained neoplasms (Fig. 1B–D). The foreign body was removed and the tricuspid valve was repaired. Two 4–0 Gore-Tex sutures were used as artificial chordae to reconstruct the subvalvular support system and coronary artery bypass grafting surgery was performed to treat the coronary stenosis. The embolism in the right lower lung was not removed because there were no obvious respiratory symptoms. According to the patient's surgical history, the characteristics of the foreign body, and the same density of shadows in the right lung at CT image, we speculate that this foreign body was bone cement. The postoperative course was uneventful and the patient was discharged on the 13th day after surgery. Echocardiography before discharge showed mild reflux in the tricuspid valve and no effusion in the pericardial cavity. An echocardiograph of the patient after 49 months indicated that tricuspid regurgitation was not aggravated. The patient was given a Class II functional classification according to the New York Heart Association.

## 3 Discussion

The most common complication of vertebroplasty and PKP is cement leakage. The incidental and insignificant cement leakage into the end plate, paravertebral space, or epidural/paravertebral veins occurs in as many as 72% to 82% of patients.^[4,5]^ Through the paravertebral or epidural veins, the cement leakage into the venous system and further migrate into the right heart and pulmonary arteries. Most cases of cement leakage are considered to be subclinical problems; however, in some cases, cement leakage can be fatal. PKP is unique in that it prevents cement leakage by creating a cavity that allows the injection of more viscous cement at a lower pressure. Furthermore, surgical procedures for preventing leakage have also been highly developed.^[6–9]^ A series of risk factors that contribute to cement leakage have been reported^[10–12]^ as follows: high injection pressure with low cement viscosity, pathological fracture (osteoporotic or tumorous), large amounts of injected poly (methyl methacrylate) (PMMA), and posterior wall defects.

In the majority of patients, a pulmonary bone-cement embolism is not a clinical event. In a study comparing vertebroplasty with conservative treatment in acute osteoporotic vertebral compression fractures (Vertos II), all patients were asymptomatic at the time of diagnosis, based on findings from imaging. Asymptomatic embolisms are usually caused by small and scattered pieces of cement in the peripheral areas of the lungs without a specific lobar location.^[13,14]^ This condition can be treated conservatively using anticoagulation drugs, such as warfarin, until the foreign body epithelializes and is no longer potentially thrombogenic.^[15,16]^ Symptomatic patients with an embolism caused by cement leakage into the right atrium are commonly managed through percutaneous retrieval. However, consideration should be given to the possible complications of this procedure, including additional thrombus fragmentation and distal emboli. For patients with right ventricular involvement or perforation, surgery is the first choice of treatment.^[17]^ With tricuspid valve injury, cardiac rupture, and multiple areas of coronary stenosis, open heart surgery is the first treatment choice for this patient.

In our patient, the chest pain and chest tightness symptoms were relieved after symptomatic and anticoagulant therapy; however, the patient's myocardial enzyme levels remained elevated, the electrocardiogram showed signs of ischemia, cardiac auscultation, and was free of murmurs. Additionally, there was no positive indication of an intracardiac foreign body on the chest x-ray. These results indicated that the patient might have had coronary atherosclerotic heart disease at an early stage, and in the subsequent CT examination, foreign bodies in the heart and pulmonary vessels were found. If a patient develops chest pain with chest tightness after PKP, the clinicians must be vigilant and ensure that the patient undergoes an echocardiograph or CT examination as early as possible because the symptoms of cardiopulmonary complications that are associated with PKP are not common, even in the elderly.^[18]^

## 4 Conclusions

PKP can produce a variety of complications including cement leakage into surrounding tissues, which might be asymptomatic, or atypical symptoms might produce x-ray results that are nonconclusive and affect our judgment. Several articles have recommended that patients receive a chest x-ray after PKP to detect atypical heart and pulmonary embolisms. We suggest that a 2-dimensional echocardiograph or chest CT should be the first choice in these cases, because of the limited sensitivity of chest x-rays. A suspected cardiac embolism is a serious condition that requires immediate action, including open heart surgery, to save the patient's life.

## 5 Statement

The study was approved by the Institutional Review Board and Ethics Committee of Union Hospital, Tongji Medical College, Huazhong University of Science and Technology. Patient has provided informed consent for publication of the case.

## Author contributions

**Conceptualization:** Yu Song, Long Wu.

**Formal analysis:** Xiaofan Huang.

**Investigation:** Yu Song.

**Methodology:** Xiaofan Huang.

**Project administration:** Long Wu.

**Software:** Yu Song, Xiaofan Huang.

**Validation:** Long Wu.

**Writing – original draft:** Yu Song.

**Writing – review & editing:** Xiaofan Huang, Long Wu.
